# microRNA-23b regulates the expression of inflammatory factors in vascular endothelial cells during sepsis

**DOI:** 10.3892/etm.2015.2224

**Published:** 2015-01-28

**Authors:** MING WU, JIAN-TENG GU, BIN YI, ZHONG-ZHI TANG, GUO-CAI TAO

**Affiliations:** 1 Department of Anesthesiology, Southwest Hospital, Third Military Medical University, Chongqing 400038, P.R. China; 2 Department of Emergency, Wuhan General Hospital of Guangzhou Military Command, Wuhan, Hubei 430070, P.R. China

**Keywords:** miR-23b, vascular endothelial cell, sepsis, nuclear factor-κB, tumor necrosis factor-α, interleukin-6, intercellular adhesion molecule-1, E-selectin, vascular cell adhesion molecule-1

## Abstract

miR-23b is a multifunctional microRNA that contributes to the regulation of multiple signaling pathways. It has been reported that miR-23b prevents multiple autoimmune diseases through the regulation of inflammatory cytokine pathways. In addition, the function and underlying mechanisms of miR-23b on sepsis are currently being investigated. In the present study, miR-23b inhibitor and mimics sequences were transfected into human vascular endothelial cells to inhibit and upregulate the expression of miR-23b, respectively. In addition, respective negative control (NC) sequences were transfected. The expression of miR-23b was found to be downregulated in the cells transfected with the mimics NC or inhibitor NC sequences following stimulation with lipopolysaccharide (LPS; P<0.01); however, higher expression levels were maintained in the cells transfected with the mimics sequence and very low levels were observed in the cells transfected with the inhibitor sequence. In addition, the expression levels of nuclear factor (NF)-κB, tumor necrosis factor (TNF)-α, interleukin (IL)-6, intercellular adhesion molecule (ICAM)-1, E-selectin and vascular cell adhesion molecule (VCAM)-1 were shown to increase following induction by LPS in the cells transfected with inhibitor/mimics NC sequences (P<0.05). However, the expression levels of these inflammatory factors decreased in the cells transfected with the mimics sequence, and increased to a greater degree in the cells transfected with the inhibitor sequence, as compared with the inhibitor NC sequences (P<0.05). Therefore, miR-23b may play a significant role in the pathogenesis and progression of sepsis by inhibiting the expression of inflammatory factors, including NF-κB, TNF-α, IL-6, ICAM-1, E-selectin and VCAM-1.

## Introduction

Sepsis is a systemic inflammatory reaction syndrome that is caused by infectious factors, and is one of the major causes of mortality in critical patients. Deregulation of the body during sepsis is considered to result in uncontrolled inflammation, the release of large amounts of inflammatory mediators, the development of inflammatory cascades and ultimately damage to tissues and organs (1–4). Vascular endothelial cells (VECs) serve as a vital interface between the blood and tissues. In the presence of inflammatory stimulation, the cells are activated and express adhesion molecules that play a critical role in leukocyte aggregation (5). Previous studies have shown that VECs are the major victim to pathogens and their toxins in sepsis. For instance, endotoxin and other bacterial components act on VECs to reduce vascular tension, widen the space between the VECs, increase vascular permeability, promote the release of inflammatory mediators and aggravate platelet aggregation (6). As a result, the inflammatory and coagulation systems become deregulated and systemic inflammatory response syndrome and multiple organ dysfunction syndrome develop (7,8). The nuclear factor (NF)-κB signaling pathway plays an important regulatory role in sepsis (9,10), and blocking the NF-κB pathway is an important modality in the treatment of sepsis (11,12).

microRNA (miRNA) is a small, single-stranded RNA molecule that is ubiquitously present in eukaryotic organisms, which is characterized by high conservation and tissue specificity. miRNA binds to specific mRNA molecules to inhibit the expression of target genes or degrade the mRNA, which subsequently contributes to cell proliferation, differentiation, development, metabolism, apoptosis and other physiological activities. Thus, miRNA exerts an important regulatory function on eukaryotic genes (13–15).

miR-23b is a multifunctional miRNA that contributes to the regulation of multiple signaling pathways, affecting cell proliferation, differentiation, apoptosis and adhesion (16–24). Moreover, the functions and underlying mechanisms are currently under investigation. It has been reported that miR-23b prevents multiple autoimmune diseases through the regulation of inflammatory cytokine pathways, in which the molecule regulates a number of inflammatory cytokines, such as NF-κB, tumor necrosis factor (TNF)-α, interleukin (IL)-1β and IL-17 (25,26). Therefore, it was hypothesized that miR-23b may act on sepsis through the NF-κB pathway and IL-17; thus, regulating the NF-κB-mediated activation of VECs.

In the present study, septic VECs were simulated using bacterial lipopolysaccharide (LPS) to induce the activation of human VECs, after which the cells were transfected with miR-23b mimics and inhibitor sequences to observe the effect of upregulating and inhibiting miR-23b on the expression levels of inflammatory factors in septic VECs. The aim of the present study was to investigate the potential of miR-23b as a therapeutic target for sepsis treatment.

## Materials and methods

### Cell culture and miR-23b sequences

The 1D3 human VEC cell line (Shanghai Bioleaf Biotech Co., Ltd., Shanghai, China) was preserved in liquid nitrogen in the laboratory. The cells were routinely cultured in modified RPMI-1640 medium containing 10% fetal bovine serum (FBS; Hyclone; GE Healthcare, Logan, UT, USA). The following sequences were designed and synthesized by Shanghai GenePharma Co., Ltd (Shanghai, China): miR-23b inhibitor sequence, 5′-GGUAAUCCCUGGCAAUGUGAU-3′; miR-23b inhibitor negative control (NC) sequence, 5′-CAGUACUUUUGUGUAGUACAA-3′; miR-23b mimics sequence, 5′-AUCACAUUGCCAGGGAUUACC-3′; miR-23b mimics NC sequence, 5′-UUCUCCGAACGUGUCACGUTT-3′. The sequences were labeled with fluorescein amidite to observe fluorescence.

### Transfection of miR-23b into the human VECs

Using Lipofectamine 2000 transfection reagent (Invitrogen Life Technologies, Carlsbad, CA, USA), the synthesized sequences were transfected into the human VECs. Initially, the mimics NC or inhibitor NC sequences were used to investigate the optimum conditions for transfection. At day one prior to transfection, 1×10^4^–3×10^4^ cells were inoculated into 24-well plates, and 500 μl modified RPMI-1640 medium containing 10% FBS was added to each well. The cells were cultured in an incubator containing 5% CO_2_ at 37°C until the cells reached a confluence of 70–90%. Various doses of mimics NC or inhibitor NC (6, 15, 20, 30, 50 or 100 pmol) were added to 50 μl serum-free Opti-MEM (Hyclone; GE Healthcare), which was followed by gentle mixing. Lipofectamine 2000 (0.3 or 1 μl) was added to 50 μl serum-free Opti-MEM, mixed gently and rested at room temperature for 5 min. The two solutions were subsequently mixed and added to the plate wells containing the cells and 500 μl serum-free RPMI-1640 medium, after which the plates were placed onto a swing bed for gentle shaking. Following incubation for 5 h at 37°C, the medium was replaced with 500 μl fresh modified RPMI-1640 medium containing serum and the plates were swung for mixing. After a further 24 h incubation at 37°C, the cells were observed and photographed under fluorescence microscopy (CX41-32RFL; Olympus, Tokyo, Japan). Pilot studies identified the optimal transfection dose of the miR-23b sequence to be 50 pmol and the optimal dose of Lipofectamine 2000 to be 1 μl, according to fluorescence intensity measurements. Therefore, the dose of the miR-23b inhibitor, NC inhibitor, miR-23b mimics and NC mimics sequences was 50 pmol, and 1 μl Lipofectamine 2000 was used for transfection. The procedure for transfection was applied as aforementioned. In addition, a blank control group was established by adding the same volume of phosphate-buffered saline and Lipofectamine 2000.

### LPS-stimulated human VECs

In total, 2×10^4^–5×10^4^ cells were successfully transfected with miR-23b and inoculated into 12-well plates. Into each well, 500 μl modified RPMI-1640 medium, containing 10% FBS, was added. The cells were cultured for 24 h at 37°C in an incubator containing 5% CO_2_. Upon reaching a confluence of 70–90%, LPS was added to a final concentration of 1 μg/ml. The cells were cultured for 4 and 8 h, after which the total RNA was extracted for quantitative polymerase chain reaction (PCR).

### Quantitative PCR

Total RNA extraction was performed according to the instructions of the TRIzol reagent kit (Invitrogen Life Technologies). Reverse transcription of the mRNA was conducted using a RevertAid First Strand cDNA Synthesis kit (Thermo Fisher Scientific, Waltham, MA, USA). The reaction system included 2 μl RNA and 1 μl oligo (dT)18 primer, and the final volume was adjusted to 12 μl with RNase-free deionized water. The solution was incubated at 70°C for 5 min on a PikoReal PCR amplifier (Thermo Fisher Scientific), and placed on ice for quick cooling. Next, 4 μl 5X buffer, 2 μl dNTP (10 mM), 1 μl RNA inhibitor and 1 μl reverse transcriptase (Thermo Fisher Scientific) were added to the solution, which was followed by mixing using a pipette. The solution was incubated at 42°C for 60 min on a PCR amplifier, followed by 5 min incubation at 70°C to inactivate the reverse transcriptase. The products of the reverse transcription reaction were quantitatively analyzed using THUNDERBIRD™ SYBR^®^ qPCR Mix kit (Toyobo Co., Ltd., Osaka, Japan). A 0.2-ml PCR tube was used, which contained 12.5 μl 2X qPCR Mix, 2.0 μl gene primer or internal standard primer (2.5 μM), 2.0 μl reverse transcripts and 8.5 μl ddH_2_O. The conditions for amplification were as follows: Initial denaturation at 95°C for 3 min, followed by 40 cycles of 95°C for 15 sec, 59°C for 30 sec and 72°C for 25 sec. The quantitative PCR assay of miR-23b was performed using a custom reverse transcription and quantitative PCR kit (Changzhou Chutian Biotechnology Co., Ltd., Changzhou, China), where U6 was used as an internal standard, according to the instructions of the kit. Table I shows the sequences of the primers used for quantitative PCR.

### Western blot analysis

Cells were harvested by centrifugation at 2,000 × g for 5 min at 37°C, and 10^6^ cells were added to 250 μl radioimmunoprecipitation assay lysis buffer (Beyotime Institute of Biotechnology, Haimen, China) to extract the total protein. In total, 50 μg protein was subjected to SDS-PAGE, after which the protein was transferred onto a 0.45-μm polyvinylidene fluoride membrane (Millipore Corporation, Billerica, MA, USA). The membrane was incubated with mouse monoclonal NF-κB P65 (1:1,000; cat. no. 13346S, Cell Signaling Technology, Inc., Danvers, MA, USA), rabbit monoclonal TNF-α (1:1,000; cat. no. ab53450), rabbit monoclonal IL-6 (1:1,000; cat. no. ab32530), rabbit monoclonal VCAM-1 (1:1,000; cat. no. ab134047, Abcam, Cambridge, UK), rabbit polyclonal ICAM-1 (1:1,000; cat. no. sc-7891), rabbit polyclonal E-selectin (1:1,000; cat. no. sc-14011) and rabbit polyclonal β-actin antibodies (1:1,000; cat. no. sc-130656, Santa Cruz Biotechnology, Inc., Santa Cruz, CA, USA), and shaken overnight at 4°C. Following discoloration, a horseradish peroxidase-conjugated goat anti-rabbit or goat anti-mouse secondary antibody (1:5,000; KPL, Inc., Gaithersburg, MD, USA) was added and incubated at room temperature for 30 min to wash out the unbound antibodies. Color development was performed by adding an enhanced chemiluminescence developer (Pierce Biotechnology, Inc., Rockford, IL, USA) and waiting for 1–2 min. Thereafter, the remnant liquid was discarded, and the membrane was embedded with a fresh membrane and subjected to X-ray exposure. The exposure condition was adjusted according to the light intensity. Following development and fixation, the images were analyzed for grayscale using BandScan 5.0 software (Glyko, Novato, CA, USA).

### Statistical analysis

All experiments were performed three times, and data are expressed as the mean ± standard deviation. A χ^2^ or two-sided t-test was performed for statistical analysis using SPSS 16.0 software (SPSS, Inc., Chicago, IL, USA). P<0.05 was considered to indicate a statistically significant difference.

## Results

### Transfection of the miR-23b inhibitor sequence inhibits miR-23b expression, while transfection of the miR-23b mimics sequence increases miR-23b expression in human VECs

**Figure 1:**
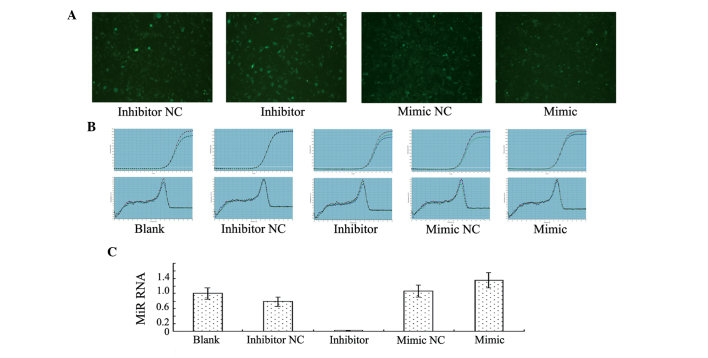
Transfection with the miR-23b inhibitor sequence inhibited miR-23b expression, while transfection with the miR-23b mimics sequence increased miR-23b expression in the human vascular endothelial cells (VECs). (A) Under fluorescence microscopy, the human VECs transfected with miR-23b emitted green fluorescence (magnification, ×100). (B and C) Fluorescence quantitative polymerase chain reaction assays revealed that the miR-23b inhibitor sequence significantly inhibited miR-23b expression; however, the miR-23b mimics sequence promoted miR-23b expression. NC, negative control.

Human VECs transfected with miR-23b emitted green fluorescence under fluorescence microscopy (Fig. 1A). The quantitative PCR results indicated that miR-23b expression decreased significantly in the group transfected with the miR-23b inhibitor sequence when compared with the blank control group and the group transfected with the inhibitor NC sequence, demonstrating that transfection with the miR-23b inhibitor inhibited miR-23b expression effectively. By contrast, miR-23b expression increased significantly in the group transfected with the miR-23b mimics sequence when compared with the blank control group and the mimics NC group, indicating that transfection with miR-23b mimics promoted miR-23b expression (Fig. 1B and C).

### LPS downregulates miR-23b expression in human VECs

**Figure 2:**
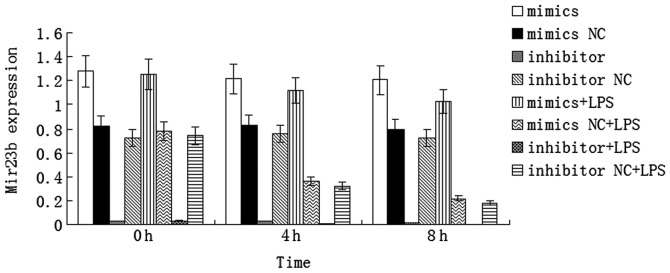
miR-23b expression decreased significantly in the human vascular endothelial cells (VECs) transfected with the mimics NC or inhibitor NC sequences at 4 or 8 h after LPS stimulation when compared with the human VECs not stimulated with LPS (P<0.01). miR-23b expression remained high in the human VECs transfected with the mimics sequence following LPS stimulation and remained very low in the cells transfected with the inhibitor sequence following LPS stimulation. NC, negative control; LPS, lipopolysaccharide.

VECs in sepsis were simulated using LPS-stimulated human VECs. The results demonstrated that miR-23b expression decreased significantly in the cells transfected with the mimics NC or inhibitor NC sequences at 4 or 8 h after LPS stimulation when compared with the cells that did not undergo LPS stimulation (P<0.01). In addition, the results revealed that miR-23b expression decreased in the LPS-stimulated human VECs; thus, VEC activation in sepsis was accompanied by the inhibition of miR-23b expression. By contrast, miR-23b expression decreased slightly in the cells transfected with the mimics sequence at 4 or 8 h after LPS stimulation when compared with the unstimulated cells, but remained at a high level. miR-23b expression remained low in the cells transfected with the inhibitor sequence following LPS stimulation (Fig. 2).

### LPS promotes inflammatory cytokine expression in human VECs

**Figure 3:**
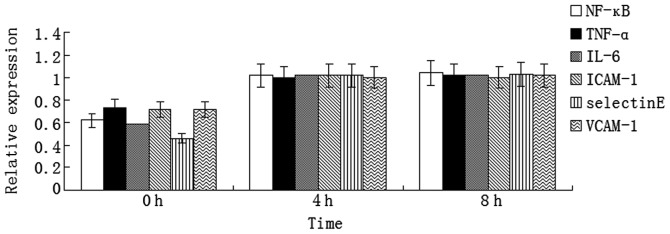
mRNA expression levels of the inflammatory factors increased significantly in the human vascular endothelial cells after 4 and 8 h of LPS stimulation (P<0.05). NF, nuclear factor; TNF, tumor necrosis factor; IL, interleukin; ICAM, intercellular adhesion molecule; VCAM, vascular cell adhesion molecule.

Expression levels of inflammatory cytokines, including NF-κB, TNF-α, IL-6, ICAM-1, E-selectin and VCAM-1, increased significantly at 4 and 8 h after LPS stimulation in the human VECs (P<0.05, Fig. 3). These results demonstrated that LPS stimulated the human VECs to express inflammatory cytokines; thus, contributing to the inflammatory reaction in sepsis.

### miR-23b mimics inhibits the LPS-stimulated expression of inflammatory factors

**Figure 4:**
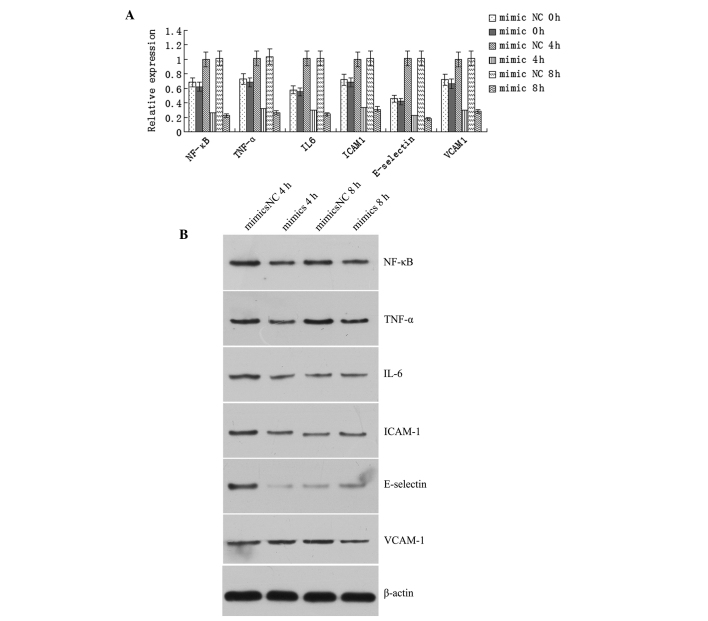
Transfection with the miR-23b mimics sequence inhibited the lipopolysaccharide (LPS)-stimulated expression of inflammatory factors. (A) Quantitative polymerase chain reaction revealed that mRNA expression levels of NF-κB, TNF-α, IL-6, ICAM-1, E-selectin and VCAM-1 increased significantly in the human vascular endothelial cells transfected with the mimics NC after 4 and 8 h of LPS stimulation, and decreased significantly in the cells transfected with the mimics sequence (P<0.05). (B) Western blot analysis revealed that the protein expression levels of NF-κB, TNF-α, IL-6, ICAM-1 and E-selectin were significantly lower in the mimics group when compared with the mimics NC group after 4 h of LPS stimulation (P<0.05). Furthermore, the protein expression level of VCAM-1 was significantly lower in the mimics group when compared with the mimics NC group after 8 h of LPS stimulation (P<0.05). NC, negative control; NF, nuclear factor; TNF, tumor necrosis factor; IL, interleukin; ICAM, intercellular adhesion molecule; VCAM, vascular cell adhesion molecule.

The mRNA expression levels of NF-κB, TNF-α, IL-6, ICAM-1, E-selectin and VCAM-1 increased significantly at 4 and 8 h after LPS stimulation in the human VECs transfected with the mimics NC sequence (P<0.05); however, the expression levels decreased significantly in the cells transfected with the mimics sequence (P<0.05; Fig. 4A). Western blot analysis revealed that the protein expression levels of NF-κB, TNF-α, IL-6, ICAM-1 and E-selectin were significantly lower in the mimics group when compared with the mimics NC group after 4 h of LPS stimulation (P<0.05), while the expression level of VCAM-1 was significantly lower in the mimics group when compared with the mimics NC group following 8 h of LPS stimulation (P<0.05; Fig. 4B).

### Effect of the miR-23b inhibitor sequence on the expression of inflammatory factors in LPS-stimulated cells

**Figure 5:**
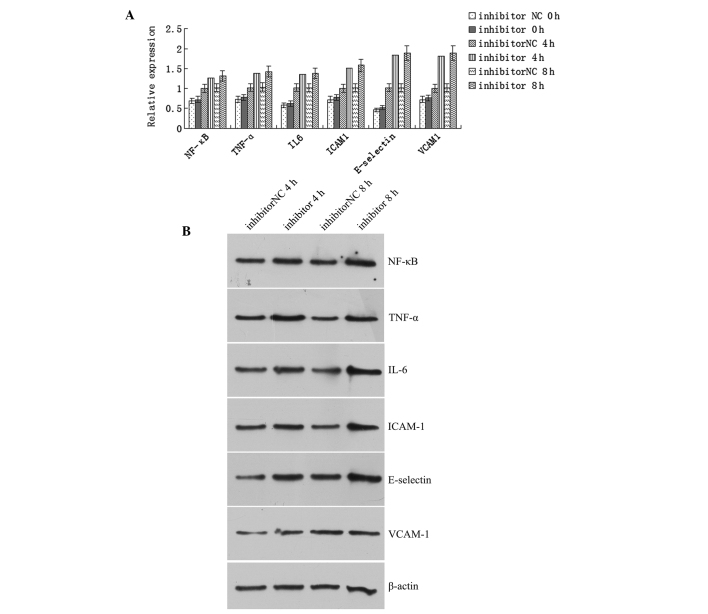
Transfection with the miR-23b inhibitor sequence promoted the expression of the inflammatory factors in lipopolysaccharide (LPS)-stimulated cells. (A) mRNA expression levels of NF-κB, TNF-α, IL-6, ICAM-1, E-selectin and VCAM-1 increased significantly in the human vascular endothelial cells transfected with the inhibitor NC after 4 and 8 h of LPS stimulation when compared with the prestimulation levels (P<0.05). In addition, expression levels of these factors increased significantly in the cells transfected with the miR-23b inhibitor sequence following LPS stimulation when compared with the prestimulation levels (P<0.05). The expression levels of these factors were greater in the cells transfected with miR-23b inhibiter as compared with those transfected with the inhibitor NC after 4 or 8 h of LPS stimulation. (B) Western blot analysis revealed that the protein expression levels of NF-κB, TNF-α, IL-6, ICAM-1, E-selectin and VCAM-1 increased significantly after 4 or 8 h of LPS stimulation in the cells transfected with the inhibitor sequence when compared with the cells transfected with the inhibitor NC (P<0.05). NC, negative control; NF, nuclear factor; TNF, tumor necrosis factor; IL, interleukin; ICAM, intercellular adhesion molecule; VCAM, vascular cell adhesion molecule.

The mRNA expression levels of NF-κB, TNF-α, IL-6, ICAM-1, E-selectin and VCAM-1 increased significantly in the human VECs transfected with the inhibitor NC sequence following LPS stimulation for 4 and 8 h when compared with the prestimulation levels (P<0.05). In addition, the expression levels of the inflammatory factors increased significantly in the cells transfected with the inhibitor sequence following LPS stimulation, as compared with the prestimulation levels (P<0.05). Furthermore, the levels of the inflammatory factors increased significantly after 4 or 8 h of LPS stimulation when compared with the levels in the cells transfected with the inhibitor NC sequence (Fig. 5A). Western blot analysis revealed that the protein expression levels of NF-κB, TNF-α, IL-6, ICAM-1, E-selectin and VCAM-1 increased significantly in the cells transfected with the inhibitor sequence when compared with the cells transfected with the inhibitor NC sequence after 4 or 8 h of LPS stimulation (P<0.05; Fig. 5B).

## Discussion

Sepsis is a systemic inflammatory reaction syndrome caused by infection, which may lead to shock and multiple organ dysfunction (27). The pathogenesis of sepsis is extremely complex (28). The invasion of pathogens triggers the release of inflammatory factors, which results in systemic inflammatory reactions. Currently, the role of miRNA in sepsis has become a focus of research. miRNA, a type of endogenous, non-encoding, single-stranded RNA with a length of ~22 nucleotides, can degrade mRNA or inhibit translation, subsequently regulating gene expression at the post-transcriptional level (29). There are a number of target genes of miRNA, with evidence demonstrating that miRNA regulates the expression of ≥30% of human genes (30,31). Upon invasion of pathogenic microorganisms, host cells have been demonstrated to produce miRNA quickly, promoting the release of inflammatory factors to cause immune hyperactivity, and inducing apoptosis or degrading the inflammatory factors to cause immunosuppression (32–37). Therefore, miRNA plays a critical role in regulating inflammatory reactions in sepsis (38). VECs are single-layer squamous cells that cover the vascular lining. The cells serve as a barrier, and are involved in substance exchange, vascular tension regulation, coagulation and the inflammatory reaction. VEC damage is closely associated with sepsis. Pathogenic microorganisms and their products may impair VECs and lead to microcirculation disturbance (39) and the amplification of inflammatory reactions (40,41). In the present study, LPS-stimulated human VECs were used to simulate VECs in sepsis. LPS is the major cell wall component of Gram-negative bacilli; thus, LPS is a major pathogenic substance that causes toxic reactions. Therefore, LPS is often used to reproduce sepsis models (42). The results demonstrated that LPS promoted the human VECs to express various inflammatory factors, including NF-κB, TNF-α, IL-6, ICAM-1, E-selectin and VCAM-1 (Fig. 2).

miR-23b is a newly identified miRNA. The molecule has been shown to exert a similar function to an oncogene in glioma, urinary system tumors (kidney cancer and prostatic carcinoma) and breast cancer (43–48). miR-23b regulates cytoskeletal reconstruction, cell invasion and metastasis (49). In addition, miR-23b has been shown to inhibit inflammatory reactions in autoimmune diseases; thus, can exert protection against autoimmune diseases (50,51). However, to the best of our knowledge, there have been no previous studies investigating the role of miR-23b in sepsis. The present study demonstrated that miR-23b expression decreased in LPS-stimulated human VECs. In order to investigate the regulatory mechanisms of miR-23b on inflammatory factor expression in VECs during sepsis, miR-23b expression in human VECs was upregulated and downregulated through transfection with miR-23b mimics and inhibitor sequences, as well as the respective NC sequences. The results demonstrated that upregulation of miR-23b inhibited the expression of NF-κB, TNF-α, IL-6, ICAM-1, E-selectin and VCAM-1, while downregulation of miR-23b promoted inflammatory factor expression.

Numerous inflammatory factors interact with each other to form a complex network, which is critical to sepsis (52,53). Previously, NF-κB has been shown to be activated in sepsis, regulating apoptosis, cell growth, the stress reaction, the immune reaction and septic shock (54–56). In addition, the levels of TNF-α and IL-6 have been shown to reach a peak as early as 3 h after the onset of sepsis, and the degree of increase may reflect the severity of sepsis (57,58). ICAM-1, VCAM-1 and E-selectin are important cell adhesion factors that regulate the activity of inflammatory and vascular endothelial cells, pro- and anti-inflammatory factors, as well as inflammatory cell migration to tissues and organs; thus, these factors play an important role in sepsis (59–61).

In conclusion, the present study demonstrated that miR-23b regulates sepsis through inhibiting the expression of inflammatory factors in VECs. The affected inflammatory factors include NF-κB, TNF-α, IL-6, ICAM-1, E-selectin and VCAM-1. These results suggest that miR-23b may be a potential therapeutic target for the treatment of sepsis. However, the present study only observed the regulation of inflammatory factors by miR-23b, however the underlying mechanisms require further study.

## Figures and Tables

**Table I:** Quantitative polymerase chain reaction primers.

Gene	Primers (5′→3′)	Length (bp)
NF-κB (NM_003998, NM_001165412)	Sense, TGAGTCCTGCTCCTTCCAA Antisense, GAGAGGTGGTCTTCACTGGG	150
IL-6 (NM_000600)	Sense, AAGCAGCAAAGAGGCACTG Antisense, TTTCACCAGGCAAGTCTCCT	106
TNF-α (NM_000594)	Sense, GTGCTGGCAACCACTAAGAAT Antisense, GCCTAAGGTCCACTTGTGTCA	170
VCAM-1(NM_001078)	Sense, GCTGCTCAGATTGGAGACTCA Antisense, CGCTCAGAGGGCTGTCTATC	100
E-selectin (NM_000450)	Sense, AATCCAGCCAATGGGTTCG Antisense, GCTCCCATTAGTTCAAATCCTTCT	104
ICAM-1 (NM_000201)	Sense, GCTCAAGTGTCTAAAGGATGGC Antisense, CATTATGACTGCGGCTGCTA	196

NF, nuclear factor; IL, interleukin; TNF, tumor necrosis factor; ICAM, intercellular adhesion molecule; VCAM, vascular cell adhesion molecule.
